# Optical coherence encryption with structured random light

**DOI:** 10.1186/s43074-021-00027-z

**Published:** 2021-04-20

**Authors:** Deming Peng, Zhaofeng Huang, Yonglei Liu, Yahong Chen, Fei Wang, Sergey A. Ponomarenko, Yangjian Cai

**Affiliations:** 1 School of Physical Science and Technology, Soochow University, Suzhou, 215006 China; 2 Shandong Provincial Engineering and Technical Center of Light Manipulation & Shandong Provincial Key Laboratory of Optics and Photonic Device, School of Physics and Electronics, Shandong Normal University, Jinan, 250014 China; 3 Department of Electrical and Computer Engineering, Dalhousie University, Halifax, Nova Scotia, B3J 2X4 Canada; 4 Department of Physics and Atmospheric Science, Dalhousie University, Halifax, Nova Scotia, B3H 4R2 Canada

**Keywords:** Structured random light, Spatial coherence, Optical encryption, Atmospheric turbulence

## Abstract

Information encryption with optical technologies has become increasingly important due to remarkable multidimensional capabilities of light fields. However, the optical encryption protocols proposed to date have been primarily based on the first-order field characteristics, which are strongly affected by interference effects and make the systems become quite unstable during light–matter interaction. Here, we introduce an alternative optical encryption protocol whereby the information is encoded into the second-order spatial coherence distribution of a structured random light beam via a generalized van Cittert–Zernike theorem. We show that the proposed approach has two key advantages over its conventional counterparts. First, the complexity of measuring the spatial coherence distribution of light enhances the encryption protocol security. Second, the relative insensitivity of the second-order statistical characteristics of light to environmental noise makes the protocol robust against the environmental fluctuations, e.g, the atmospheric turbulence. We carry out experiments to demonstrate the feasibility of the coherence-based encryption method with the aid of a fractional Fourier transform. Our results open up a promising avenue for further research into optical encryption in complex environments.

## Introduction

As a fundamental attribute that describes statistical properties of random light fields, optical coherence has played an important role in understanding and tailoring light-matter interactions [1, 2]. Spatial coherence, in particular, governs a multitude of intriguing optical phenomena and features in a number of applications [3, 4]. Even a straightforward adjustment of a spatial coherence length of light can go a long way to improve the image quality of a microscope [5, 6] and to reduce the turbulence induced field degradation in optical communications [7]. The early theoretical [8, 9] and experimental [10] work on light beams with nonuniform spatial coherence has triggered research into engineering spatial coherence structure of random sources. This initial interest has been further invigorated by the work of Gori and coauthors [11, 12] who have established a general framework for generating random beams with statistically uniform (Schell-model) [13–19] and nonuniform [20–27] spatial coherence distributions. The spatial coherence structure engineering at the source has been shown to endow optical fields with a number of nontrivial features, such as diffraction-free propagation [9, 24], efficient self-healing [28, 29], self-focusing [20, 30, 31], self-steering [32, 33], and self-shaping [34, 35] capabilities as well as periodicity reciprocity [36, 37]. A rich repertoire of propagation scenarios induced by the spatial coherence engineering enables a host of promising applications to photovoltaics [38], diffractive imaging with low-coherence light [39], optical target tracking [40, 41], and particle trapping [42, 43] among others.

At the same time, information encryption with the aid of optical technologies has been studied extensively in the past decade due to remarkable multidimensional capabilities and ultrafast modulation speed afforded by the light fields [44, 45]. Numerous protocols for optical information encryption have been proposed since the double random phase encoding (DRPE) was developed by Réfrégier and Javidi [46]. Much research on optical information encryption has been carried out to date using the DRPE or DRPE related techniques, including a fractional Fourier domain DRPE [47], lensless DRPE in the Fresnel domain [48], and multidimensional random phase encoding [49], among others. Recent progress in the light field structure engineering [50–52], has highlighted the degrees of freedom of a structured light field as powerful tools for information encoding. For example, the optical encryption protocols based on the phase structure modulation [53] and orbital angular momentum and polarization state mode division multiplexing [54–60] have been developed lately.

To the best of our knowledge, however, the optical encryption protocols proposed thus far have been primarily based on the modulation of the first-order characteristics of *coherent* optical fields, such as the phase, amplitude, and polarization of light. The latter are quite sensitive to any interference effects inevitably arising during light propagation and its interaction with the matter [61]. On the other hand, the second-order correlations of *statistical* light fields [1] are known to be fairly robust to environmental noise such as the atmospheric turbulence, especially in the low coherent limit [7, 62]. This observation begs a natural question: Can any information be encrypted into the spatial coherence structure of a statistical light field and robustly transmitted through a hostile environment?

In this work, we propose an efficient protocol for the encryption of optical image information into the spatial coherence structure of a random beam with the help of a generalized van Cittert–Zernike theorem involving the encoding system with encryption keys. We argue that the measurement of the spatial coherence structure to decode the information enhances the protocol security because the measurement of a statistical characteristic requires collecting much more data than is the case for acquiring deterministic quantities employed in the traditional optical encryption protocols. Moreover, we demonstrate experimentally that the advanced protocol is extremely robust to the environmental fluctuations, such that the information can be well reconstructed despite the atmospheric turbulence of any strength. Our results pave a way toward robust optical coherence encryption in complex noisy environments.

## Method

### Principle of coherence-based encryption

**Fig. 1:**
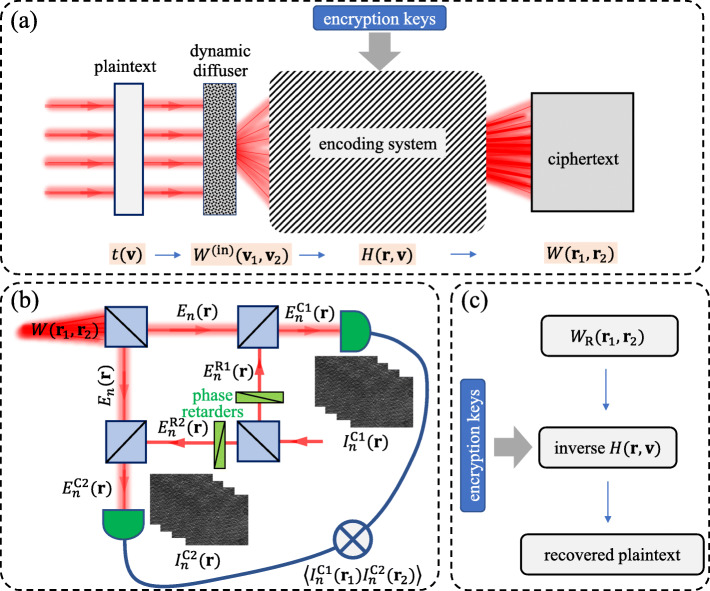
Principle of optical coherence-based encryption. **a** Encoding of an optical image with a transmission function *t*(**v**) (plaintext) into the spatial coherence structure *W*(**r**_1_,**r**_2_) (ciphertext) of a random light beam through a generalized van Cittert–Zernike theorem formed by a dynamic diffuser and an encoding system with the encryption keys embeded inside. **b** The generalized Hanbury–Brown Twiss experiment setup for measuring the complex spatial coherence structure. The phase difference between the reference fields is controlled by the phase retarders. **c** Decoding of the plaintext from the measured spatial coherence structure *W*_R_(**r**_1_,**r**_2_) and the encryption keys via an inverse transfer function of the encoding system

We schematically represent an optical encryption protocol based on the spatial coherence structure engineering in Fig. 1. The proposed protocol consists of three stages. At the first stage, a plaintext (i.e., an amplitude image) is encoded into the spatial coherence structure of a random light beam via a generalized van Cittert–Zernike theorem. Next, the spatial coherence structure of the beam, containing a ciphertext, is measured through a generalized Hanbury–Brown Twiss experiment. Finallly, the information encoded into the plaintext is decoded by using the measured spatial coherence structure and the encryption keys involved in the encoding system shown in part (a) to Fig. 1.

#### Encoding

In Fig. 1a we display the encoding stage of our protocol. The plaintext information, i.e., an image with its (real) transmission function *t*(**v**) is first embedded into a completely incoherent light source by transmitting coherent light through a dynamic diffuser, e.g., a rotating ground-glass disk (RGGD). The characteristic inhomogeneity scale is much smaller than the beam spot size on its surface [61]. The second-order correlations of the light field generated by the incoherent source in the space-frequency domain can be characterized by the cross-spectral density as

1 $$  W^{(\text{in})}(\mathbf{v}_{1}, \mathbf{v}_{2}) = t(\mathbf{v}_{1})t(\mathbf{v}_{2}) \delta(\mathbf{v}_{1} - \mathbf{v}_{2}),  $$

where **v**_1_ and **v**_2_ are two arbitrary position vectors in the source plane, *δ*(·) is a Dirac delta function and we drop the frequency dependence of *W* hereafter, for brevity. We then transform the incoherent light field into a partially coherent beam with the help of an optical system with a transfer function *H*(**r**,**v**). The cross-spectral density of the partially coherent beam then reads

2 $$  W(\mathbf{r}_{1}, \mathbf{r}_{2}) = \iint W^{(\text{in})}\left(\mathbf{v}_{1}, \mathbf{v}_{2}\right) H^{\ast}(\mathbf{r}_{1}, \mathbf{v}_{1})H(\mathbf{r}_{2}, \mathbf{v}_{2}) \mathrm{d}^{2} \mathbf{v}_{1} \mathrm{d}^{2} \mathbf{v}_{2},  $$

where **r**_1_ and **r**_2_ are two arbitrary position vectors in the output plane of the optical system and the asterisk denotes a complex conjugate.

We call the transform in Eq. (2) a generalized van Cittert–Zernike theorem as the original one describes the evolution of the cross-spectral density of light, generated by an extended incoherent source, on free space propagation [1]. The optical imaging system that we employ does not merely consist of simple lenses and stretches of free space. Rather, it is, in general, a complicated system containing the encryption keys. We remark here that our protocol can accommodate any encoding system of the type previously employed in optical encryption protocols with coherent light. The encryption systems characterized by linear and nonlinear transfer functions will give rise to random light beams with statistically uniform (Shell-model) [15] and nonuniform [27] spatial correlations, respectively. Similar to the case of conventional, coherent encryption protocols, the complexity of the encoding protocol ensures the encoded data security. The attack analysis for different types of encryption protocols was previously studied elsewhere [63, 64] and will not be addressed here.

The difference between the traditional encoding protocols and our coherence-based one is that in our case, given plaintext information is encoded into second-order field correlations rather than into a deterministic first order characteristic, such as light field amplitude, phase, or polarization. As a consequence, the information about an entire statistical ensemble of a large number of field realizations is required to reconstruct such field correlations, making the information concealed in the ciphertext hard to compromise. Furthermore, a relative insensitivity of the second-order field correlations to the environmental fluctuations augur well for the robustness of our protocol in noisy environments.

#### Ciphertext measurement

The spatial coherence of a partially coherent light beam can be measured, typically, with the classic Young’s two-pinhole experiment. However, the tiny size of the pinhole opening limits the light efficiency of the measurement and the large sets of pinhole-separation data increases the measurement time. Here, to fully recover a complex-valued spatial coherence structure distribution, specified by the cross-spectral density, we apply a recently advanced generalized Hanbury–Brown Twiss technique [40, 41]. To this end, we first introduce a pair of independently controlled coherent reference fields *E*^R1^(**r**) and *E*^R2^(**r**) and combine them with the output random light beam [see Fig. 1b]. The field realizations of two composite random fields can then be written as

3 $$\begin{array}{*{20}l}  E^{\text{C1}} (\mathbf{r})& = E^{\text{R1}} (\mathbf{r})+ E(\mathbf{r}), \end{array} $$

4 $$\begin{array}{*{20}l}  E^{\text{C2}} (\mathbf{r})& = E^{\text{R2}} (\mathbf{r})+ E(\mathbf{r}), \end{array} $$

where *E*(**r**) is a field realization of the partially coherent beam which yields the cross-spectral density of the beam as *W*(**r**_1_,**r**_2_)=〈*E*^∗^(**r**_1_)*E*(**r**_2_)〉. Hereafter unless specified otherwise, the angle brackets denote ensemble (time) averaging over the field realizations.

The intensity-intensity cross-correlation of the two composite fields at any pair of points can be expressed as

5 $$\begin{array}{*{20}l}  G^{\mathrm{C}} (\mathbf{r}_{1}, \mathbf{r}_{2}) = \langle I^{\text{C1}} (\mathbf{r}_{1}) I^{\text{C2}} (\mathbf{r}_{2}) \rangle, \end{array} $$

where *I*^C1^(**r**)=*E*^C1∗^(**r**)*E*^C1^(**r**) and *I*^C2^(**r**)=*E*^C2∗^(**r**)*E*^C2^(**r**) denote the random intensities of the two composite fields. On substituting from Eqs. (3) and (4) into Eq. (5) and employing a Gaussian moment theorem [61], we obtain

6 $$\begin{array}{*{20}l}  G^{\mathrm{C}} (\mathbf{r}_{1}, \mathbf{r}_{2}) = & \langle I^{\text{U1}} (\mathbf{r}_{1}) \rangle \langle I^{\text{U2}} (\mathbf{r}_{2}) \rangle + |W(\mathbf{r}_{1}, \mathbf{r}_{2})|^{2} + 2\sqrt{I^{\text{R1}} (\mathbf{r}_{1}) I^{\text{R2}} (\mathbf{r}_{2})}  \\ & \times \left[ W^{\prime}(\mathbf{r}_{1}, \mathbf{r}_{2}) \cos \Delta \phi- W^{\prime \prime}(\mathbf{r}_{1}, \mathbf{r}_{2}) \sin \Delta \phi \right], \end{array} $$

where *I*^U1^(**r**)=*I*^R1^(**r**)+*I*(**r**) and *I*^U2^(**r**)=*I*^R2^(**r**)+*I*(**r**). Here *I*^R1^(**r**)=|*E*^R1^(**r**)|^2^ and *I*^R2^(**r**)=|*E*^R2^(**r**)|^2^ are the intensities of the two reference fields, *I*(**r**)=|*E*(**r**)|^2^ is an intensity of the random beam; prime and double prime denote, respectively, the real and the imaginary parts, and *Δϕ*=arg[*E*^R1^(**r**_1_)]−arg[*E*^R2^(**r**_2_)] is a phase difference between the two reference fields at points **r**_1_ and **r**_2_, with arg standing for the phase of a complex function. We note that the Gaussian moment theorem is applicable here since the field of a random beam is produced by an RGGD and therefore, it obeys Gaussian statistics [61].

We can infer from Eq. (6) that the information about the real and imaginary parts of the cross-spectral density function is contained in the intensity-intensity cross-correlation function *G*^C^(**r**_1_,**r**_2_). Hence, by setting the phase difference *Δϕ* to either 0 or *π*/2, we can separately extract the real or imaginary part. Further, we notice that the first two terms in Eq. (6) form irrelevant background which can be removed by first evaluating an auxiliary intensity-intensity cross-correlation as

7 $$  G^{\mathrm{U}} (\mathbf{r}_{1}, \mathbf{r}_{2}) = \langle I^{\text{U1}} (\mathbf{r}_{1}) I^{\text{U2}} (\mathbf{r}_{2}) \rangle,  $$

where *I*^U1^(**r**) and *I*^U2^(**r**) are (fluctuating) combined intensities of the reference and random beams. With the help of the Gaussian moment theorem, Eq. (7) can be written as

8 $$  G^{\mathrm{U}} (\mathbf{r}_{1}, \mathbf{r}_{2}) = \langle I^{\text{U1}} (\mathbf{r}_{1}) \rangle \langle I^{\text{U2}} (\mathbf{r}_{2}) \rangle + |W(\mathbf{r}_{1}, \mathbf{r}_{2})|^{2}.  $$

It follows from Eqs. (6) and (8) that the real and imaginary parts of the cross-spectral density can be obtained as

9 $$\begin{array}{*{20}l}  W^{\prime} (\mathbf{r}_{1}, \mathbf{r}_{2}) &= \frac{\Delta G(\mathbf{r}_{1}, \mathbf{r}_{2},\Delta \phi = 0)}{2\sqrt{I^{\text{R1}} (\mathbf{r}_{1}) I^{\text{R2}} (\mathbf{r}_{2})}}, \end{array} $$

10 $$\begin{array}{*{20}l}  W^{\prime \prime} (\mathbf{r}_{1}, \mathbf{r}_{2}) & = -\frac{\Delta G(\mathbf{r}_{1}, \mathbf{r}_{2},\Delta \phi = \pi/2)}{2\sqrt{I^{\text{R1}} (\mathbf{r}_{1}) I^{\text{R2}} (\mathbf{r}_{2})}}, \end{array} $$

where *ΔG*(**r**_1_,**r**_2_,*Δϕ*)=*G*^C^(**r**_1_,**r**_2_)−*G*^U^(**r**_1_,**r**_2_) is expressed in terms of the phase delay *Δϕ*.

It follows from Eqs. (3)–(10) that the cross-spectral density function can be fully recovered by evaluating the intensity-intensity correlations in Eqs. (5) and (7). In the experiment, the random intensities *I*^C^(**r**) and *I*^U^(**r**) of *N* realizations are recorded at different time instants and the intensity-intensity correlations are then evaluated as [17]

11 $$\begin{array}{*{20}l}  G^{\mathrm{C}} (\mathbf{r}_{1}, \mathbf{r}_{2}) &= \frac{1}{N} \sum_{n=1}^{N} I^{\mathrm{C}}_{n}(\mathbf{r}_{1}) I^{\mathrm{C}}_{n}(\mathbf{r}_{2}), \end{array} $$

12 $$\begin{array}{*{20}l}  G^{\mathrm{U}} (\mathbf{r}_{1}, \mathbf{r}_{2}) &= \frac{1}{N} \sum_{n=1}^{N} I^{\mathrm{U}}_{n}(\mathbf{r}_{1}) I^{\mathrm{U}}_{n}(\mathbf{r}_{2}), \end{array} $$

where $I^{\mathrm {C}}_{n}(\mathbf {r})$ and $I^{\mathrm {U}}_{n}(\mathbf {r})$ are intensities of the *n*th field realization. We remark that whenever a partially coherent beam is generated by a Fourier transforming system, such as a thin lens, the time average in Eqs. (5) and (7) can be replaced with the space average over a single instantaneous speckle pattern [40, 65].

#### Decoding

Once the cross-spectral density *W*(**r**_1_,**r**_2_) has been fully recovered, the plaintext can be decoded by using an inverse transform of Eq. (2) with the correct encryption keys. As is elucidated in Fig. 1c, the decoded paintext is correctly recovered only for the matched spatial coherence and encryption keys.

### Example

To demonstrate the feasibility of our encryption method, we present an example of our protocol in which the optical encryption system performs a fractional Fourier transform of a given order, c.f., [47]. The corresponding transfer function then reads

13 $$  H(\mathbf{r}, \mathbf{v}) = A \exp \left[ \frac{\mathrm{i}\pi}{\lambda f} \left(\text{cot}\varphi_{\mathrm{E}} \mathbf{v}^{2} - 2\text{csc} \varphi_{\mathrm{E}} \mathbf{v} \cdot \mathbf{r} + \text{cot} \varphi_{\mathrm{E}} \mathbf{r}^{2}\right) \right],  $$

where *A*=−icsc*φ*_E_/*λf*, *φ*_E_=*p*_E_*π*/2 and *p*_E_ is an order of the fractional Fourier transform. The magnitude of *p*_E_ can be regarded as an encryption key of the protocol. In our experiment, the fractional Fourier transform system can be realized by a thin convex lens of focal length *f*_convex_=*f*/ sin*φ*_E_, which is placed midway between the input and output planes of the encoding system. The distances between the convex lens and the input/output planes are equal to *f* tan(*φ*_*E*_/2). Further, the encryption key falls in the range: *p*_E_∈(0,1], with *p*_E_=1 corresponding to the ordinary Fourier transform limit.

It follows from Eqs. (2) and (13) that the ciphertext can the be expressed as

14 $$  W(\mathbf{r}_{1},\mathbf{r}_{2}) = A^{2} \int I_{\mathrm{E}}(\mathbf{v}) \exp \left[ \frac{\mathrm{i}\pi}{\lambda f}\left(2 \text{csc} \varphi_{\mathrm{E}}\mathbf{v}\cdot \Delta \mathbf{r} - \text{cot} \varphi_{\mathrm{E}} \mathbf{r}_{1}^{2} + \text{cot} \varphi_{\mathrm{E}} \mathbf{r}_{2}^{2}\right) \right] \mathrm{d}^{2} \mathbf{v},  $$

where *I*_E_(**v**)=*t*^2^(**v**) denotes the plaintext intensity and *Δ***r**=**r**_1_−**r**_2_. We can infer from Eq. (14) that the cross-spectral density *W*(**r**_1_,**r**_2_) is a two-point fractional Fourier transform of the image intensity *I*_E_(**v**). Thus, the image can be recovered by an inverse fractional Fourier transform as

15 $$  I_{\mathrm{R}}(\mathbf{v}) = \iint W_{\mathrm{R}}(\mathbf{r}_{1},\mathbf{r}_{2}) \exp \left[ -\frac{\mathrm{i}\pi}{\lambda f}\left(2 \text{csc} \varphi_{\mathrm{R}} \Delta \mathbf{r}\cdot \mathbf{v} - \text{cot} \varphi_{\mathrm{R}} \mathbf{r}_{1}^{2} + \text{cot} \varphi_{\mathrm{R}} \mathbf{r}_{2}^{2}\right) \right] \mathrm{d}^{2} \mathbf{r}_{1}\mathrm{d}^{2} \mathbf{r}_{2}.  $$

Here *I*_R_(**v**) is an intensity recovered from the measured cross-spectral density function *W*_R_(**r**_1_,**r**_2_) and *p*_R_ is a decoding key. Next, *φ*_R_=*p*_R_*π*/2. Only if does the measured *W*_R_(**r**_1_,**r**_2_) match the ciphertext *W*(**r**_1_,**r**_2_) and *p*_R_=*p*_E_, the plaintext intensity is correctly recovered: *I*_R_(**v**)=*I*_E_(**v**).

Notice that Eq. (15) can be viewed as a four-dimensional Fourier transform of the function $W_{\mathrm {R}}(\mathbf {r}_{1},\mathbf {r}_{2}) \exp \left [ \frac {\mathrm {i}\pi }{\lambda f}\left (\text {cot} \varphi _{\mathrm {R}} \mathbf {r}_{1}^{2} - \text {cot} \varphi _{\mathrm {R}} \mathbf {r}_{2}^{2}\right) \right ]$, which can, in turn, be regarded as the cross-spectral density of a “effective” chirped random beam. Further, it follows from Eq. (14) that such a beam can be assumed to be generated from an incoherent light source with its intensity being *I*_R_(**v**) with a Fourier transforming imaging system. Thus, per our previous discussion, the cross-spectral density of the effective chirped random beam can be recovered by using space instead of time averaging in the generalized Hanbury Brown–Twiss experiment. The corresponding reference fields read

16 $$\begin{array}{*{20}l}  E^{\text{R1} \prime} (\mathbf{r}) &= E^{\text{R1}} (\mathbf{r}) \exp\left(\frac{\mathrm{i}\pi}{\lambda f} \cot \varphi_{\mathrm{R}} \mathbf{r}^{2}\right), \end{array} $$

17 $$\begin{array}{*{20}l}  E^{\text{R2} \prime} (\mathbf{r}) &= E^{\text{R2}} (\mathbf{r}) \exp\left(\frac{\mathrm{i}\pi}{\lambda f} \cot \varphi_{\mathrm{R}} \mathbf{r}^{2}\right). \end{array} $$

Here the quadratic phase can be introduce by a concave lens. The distance between the lens and the output reference field is *d*=*f*/cot*φ*_R_−*f*_concave_, where *f*_concave_ is a focal length of the concave lens. On substituting from Eqs. (16) and (17) into Eqs. (3), (4) and (5) and taking a Gaussian average, we obtain the real and imaginary parts of the recovered cross-spectral density of the chirped random beam from Eqs. (9) and (10). Next, the plaintext can be recovered by an inverse Fourier transform of the measured cross-spectral density. We note in passing that the encryption key *p*_R_ for decoding the plaintext is now used in the chirped reference fields of Eqs. (16) and (17) that are introduced at the ciphertext interrogation stage.

**Fig. 2:**
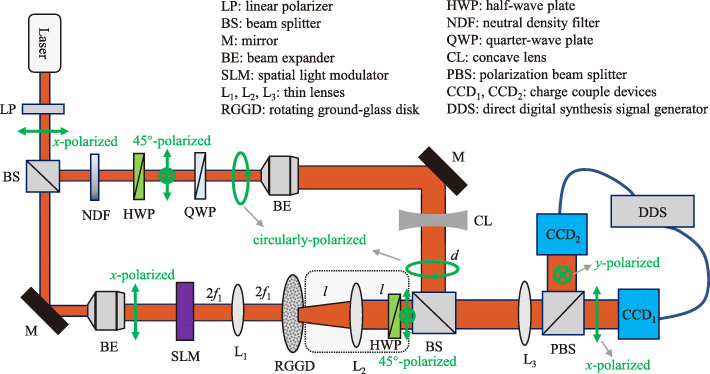
Schematics of an experimental setup for optical image encryption into the spatial coherence structure of a random light beam and a setup for measuring the corresponding cross-spectral density function. The green arrows stand for the polarization directions of the light beams

We exhibit our experimental setup in Fig. 2. We first convert an unpolarized monochromatic, coherent beam of carrier wavelength *λ*=633 nm, generated by a He–Ne laser, into an *x*-polarized beam by a linear polarizer (LP) and then split the beam by a beamsplitter (BS) into two beams that go into the top and bottom arms, respectively, as shown in Fig. 2. We employ the top arm in the figure to generate reference light beams with their electric fields given by Eqs. (16) and (17). The purpose of the bottom arm is to encode the image information into the cross-spectral density of a random beam as illustrated in Fig. 1a. In the bottom arm, we impinge the *x*-polarized beam, having been expanded by a beam expander (BE), onto a spatial light modulator (SLM) to which we preload an optical image with a transmission function *t*(**v**). We then project the outgoing SLM-modulated coherent light onto a rotating ground-glass disk (RGGD) by a 2*f* imaging system formed by a thin lens L_1_ of focal distance *f*_1_=100 mm. In our experiment, the beam spot on the RGGD is much larger than the characteristic inhomogeneity scale of the latter. Thus, the emerged light from the RGGD can be regarded as incoherent and its cross-spectral density can be well approximated by Eq. (1). The incoherent light by the secondary source is then transmitted through a fractional Fourier transform system represented by a thin lens L_2_ of focal length *f*_2_=250 mm. The random beam with the encoded into its cross-spectral density image information is thus generated in the output plane of the fractional Fourier transform system. The distance *l* between the RGGD and L_2_ (or between L_2_ and the output plane) can be varied to realize a fractional Fourier transform of any given order *p*_E_. As a part of the fractional Fourier transform system, a half-wave plate (HWP) with its fast axis making the angle *π*/8 with the *x*-polarization direction is inserted to transform the *x*-polarized random beam into a *π*/4-linearly polarized one.

In the top arm, we first transmit the *x*-polarized beam through a neutral-density filter (NDF), an HWP, and a quarter-wave plate (QWP). We set the fast axes of the HWP and QWP to make the angle *π*/8 with respect to and to be parallel to the *x*-polarization direction, respectively. Therefore, we can generate a right-handed circularly polarized coherent beam immediately past the QWP. We then expand the circularly polarized reference beam by the BE to produce a collimated beam of virtually uniform intensity distribution. Due to a fixed *π*/2 phase difference between the two orthogonal components of the circularly polarized beam, its *x*- and *y*-polarized components can be viewed as two coherent reference fields required for the measurement of the cross-spectral density of the random probe beam. We can introduce the quadratic phases of the reference fields in Eqs. (16) and (17) by adding a concave lens (CL) of focal distance *f*_concave_=100 mm behind the BE. We can adjust the quadratic phase by changing the distance *d* between the CL and the output plane of the reference beam.

We next combine the reference beam, generated in the top arm, and the random beam, generated in the bottom arm, with a BS to measure the cross-spectral density of the composite beam (i.e., the ciphertext), as illustrated in Fig. 1b. We split the *x*- and *y*-components of the composite field by a polarization beamsplitter (PBS) and image them onto CCD_1_ and CCD_2_, respectively, by a 2*f* imaging system formed by a couple of thin lens L_3_ of focal distance *f*_3_=250 mm. We use a direct-digital synthesis (DDS) signal generator as an external trigger to control the two CCDs to simultaneously capture the random intensities of the *x*- and *y*-components of the field.

In our experiment, the message in capital characters ‘SUDA’ is loaded into the SLM and the fractional order of the encryption system is set to be *p*_E_=0.6 by setting the distance to *l*=103 mm. To measure the cross-spectral density of the composite beam, we record the intensity distributions *I*_*x*_(**r**) and *I*_*y*_(**r**) of the *x*- and *y*-components of the *π*/4-linearly polarized random beam by blocking the reference arm, the intensity distributions $I^{\mathrm {R}}_{x}(\mathbf {r})$ and $I^{\mathrm {R}}_{y}(\mathbf {r})$ of the *x*- and *y*-components of the circularly polarized reference beam by blocking the encryption arm, and the intensity distributions $I^{\mathrm {C}}_{x}(\mathbf {r})$ and $I^{\mathrm {C}}_{y}(\mathbf {r})$ of the *x*- and *y*-components of the composite field. The real and imaginary parts of the recovered cross-spectral density function follow from

18 $$\begin{array}{*{20}l}  W^{\prime}_{\mathrm{R}} (\mathbf{r}_{1}, \mathbf{r}_{2}) &= \frac{\left\langle I^{\mathrm{C}}_{x}(\mathbf{r}_{1}) I^{\mathrm{C}}_{x}(\mathbf{r}_{2})\right\rangle_{\mathrm{s}}-\left\langle [I_{x}(\mathbf{r}_{1})+I^{\mathrm{R}}_{x}(\mathbf{r}_{1})] [I_{x}(\mathbf{r}_{2})+I^{\mathrm{R}}_{x}(\mathbf{r}_{2})]\right\rangle_{\mathrm{s}}}{2\sqrt{I^{\mathrm{R}}_{x} (\mathbf{r}_{1}) I^{\mathrm{R}}_{x} (\mathbf{r}_{2})}}, \end{array} $$

19 $$\begin{array}{*{20}l}  W^{\prime \prime}_{\mathrm{R}} (\mathbf{r}_{1}, \mathbf{r}_{2}) & = -\frac{\left\langle I^{\mathrm{C}}_{x}(\mathbf{r}_{1}) I^{\mathrm{C}}_{y}(\mathbf{r}_{2})\right\rangle_{\mathrm{s}}-\left\langle [I_{x}(\mathbf{r}_{1})+I^{\mathrm{R}}_{x}(\mathbf{r}_{1})] [I_{y}(\mathbf{r}_{2})+I^{\mathrm{R}}_{y}(\mathbf{r}_{2})]\right\rangle_{\mathrm{s}}}{2\sqrt{I^{\mathrm{R}}_{x} (\mathbf{r}_{1}) I^{\mathrm{R}}_{y} (\mathbf{r}_{2})}}, \end{array} $$

where 〈·〉_s_ denotes a spatial average. We can reconstruct the image *I*_R_(**v**) by evaluating an inverse Fourier transform of the measured cross-spectral density *W*_R_(**r**_1_,**r**_2_). We control the magnitude of the encryption key *p*_R_ by adjusting the distance *d* between the CL and the BS. For the correct key, i.e., *p*_R_=*p*_E_=0.6, the distance is *d*=178 mm.

## Results and discussion

**Fig. 3:**
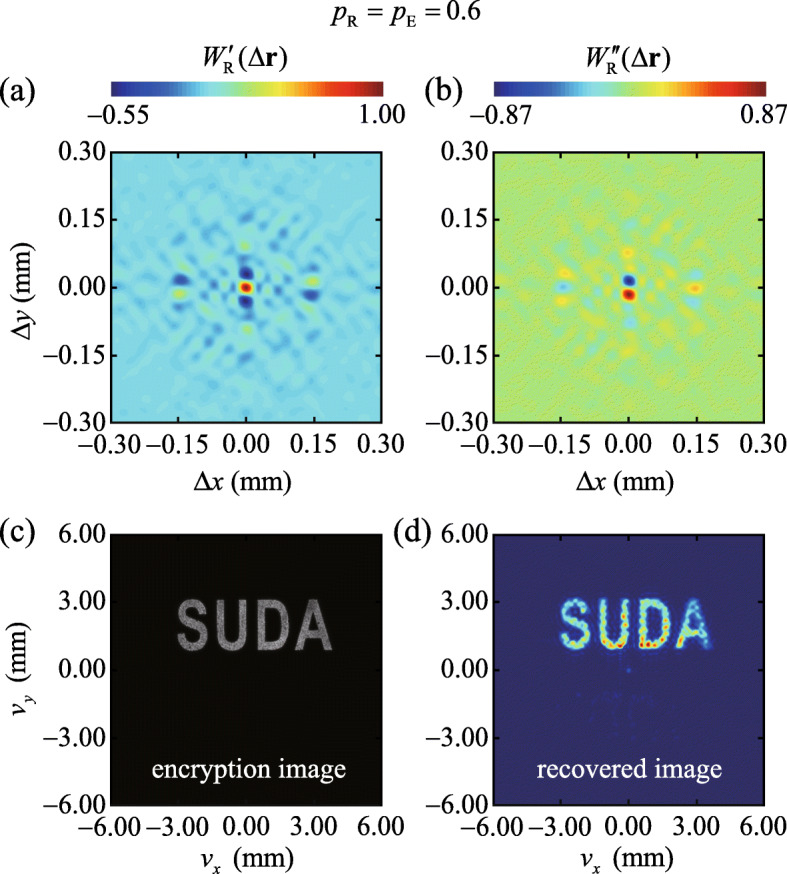
Experimental results for the real (**a**) and imaginary (**b**) parts of the recovered cross-spectral density *W*_R_(*Δ***r**) for the case *p*_R_=*p*_E_=0.6. **c** The measured intensity distribution of the encrypted image on the RGGD. **d** The reconstructed image from the Fourier transform of the measured cross-spectral density function in (**a**) and (**b**)

In Figs. 3a and b, we depict our experimental results for the measured cross-spectral density of the effective chirped random beam, corresponding to the fractional order *p*_R_ in the reference arm equal to the fractional order *p*_E_ at the encoding stage. In Fig. 3d we show the intensity *I*_R_(**v**) of a recovered image obtained with the aid of an inverse Fourier transform of the measured cross-spectral density. We also display in Fig. 3c the image illuminating the RGGD, i.e., the plaintext, for comparison. It can be inferred from Fig. 3 that the cross-spectral density of a random beam can, indeed, be viewed as an effective carrier of optically encoded information. Furthermore, we find that whenever the encryption and decoding key match well, the decoded image is consistent with the input to the system.

**Fig. 4:**
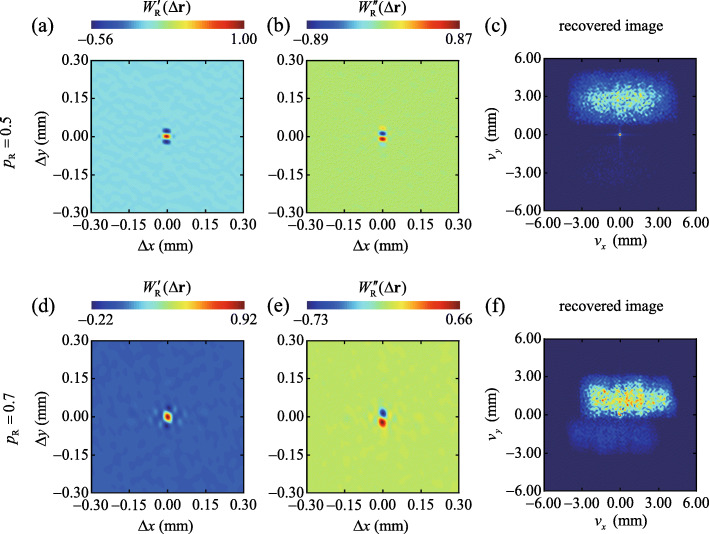
**a** through **e**. Experimental results for the real (left panels and imaginary (middle panels) parts of the recovered cross-spectral density *W*_R_(*Δ***r**) for *p*_R_=0.5 (top) and *p*_R_=0.7 (bottom), respectively. **c** and **f** Reconstructed images from a Fourier transform of the measured cross-spectral density functions

**Fig. 5:**
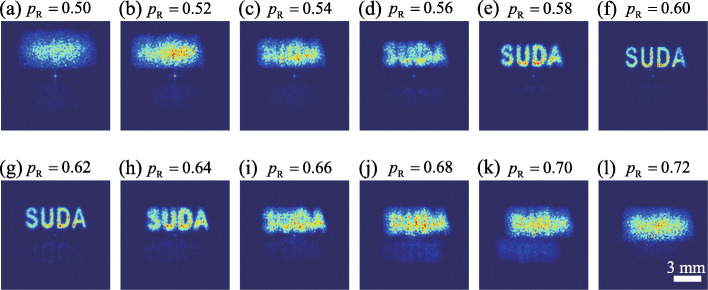
Experimental results for the reconstructed images for *p*_R_ varying from 0.5 to 0.72 with the step size of 0.02. The scale bar is shown in panel (**l**)

To verify the reliability of our encryption protocol, we vary the distance *d* between the CL and BS to introduce mismatch between *p*_R_ and *p*_E_. In Fig. 4 we exhibit the experimental results for the measured cross-spectral density and the corresponding reconstructed image for *p*_R_=0.5 (top) and *p*_R_=0.7 (bottom). We can infer from Figs. 4c and f that the original plaintext image is unrecognizable as long as *p*_R_≠*p*_E_. To visualize the evolution of the reconstructed image with *p*_R_, we show in Fig. 5 the experimental results for the reconstructed images with *p*_R_ varying from 0.5 to 0.72, with the increments of 0.02. We observe that the reconstructed image rapidly blurs as the difference between *p*_R_ and *p*_E_ grows. The plaintext image, i.e., ‘SUDA’, can only be recognized for *p*_R_=0.6±0.04. The existence of such value range is due to the property of the fractional Fourier transform function used in the encoding system. The function contains only a single encryption key. We remark that the security of the system can be further improved, e.g., by using a more complicated response function and by increasing the number of encryption keys in the encoding system [64].

**Fig. 6:**
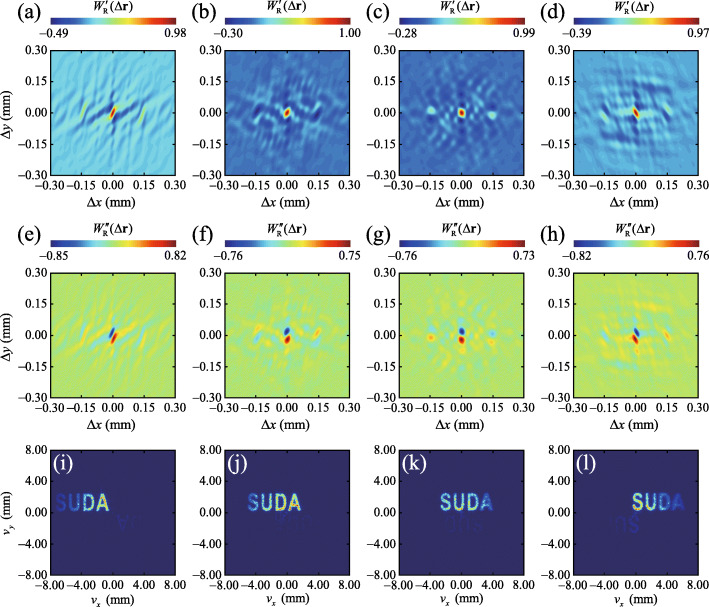
Encryption of a moving object with the modulation of the cross-spectral density function. **a**–**d** Experimental results for the real part of the measured cross-spectral density of the beam illuminating a moving object located at different instantaneous positions. **e**–**h** Experimental results for the imaginary part of the corresponding measured cross-spectral density functions. **i**–**l** Experimental results for the reconstructed images at different locations

We can infer from Eq. (14) that not only the structure but also the position of the image can be encoded into the cross-spectral density function of a random beam. It follows that our protocol allows for moving image encryption. To illustrate this point, we loaded a string of characters ‘SUDA’, moving along a straight line, into the SLM. In the top two panels of Fig. 6, we show the experimental results for the measured cross-spectral density of the beam illuminating the moving string at different locations. In the bottom panels, we display the corresponding reconstructed images. In this experiment, *p*_E_=*p*_R_=0.6. The experimental results in Fig. 6 indicate that the cross-spectral density of the illuminating beam can be successfully employed to carry information about the structure and instantaneous position of a moving image. We notice here the time scale for encryption and decryption is about 0.733 seconds, which is shorter than the time difference (1 second) between two locations of the object in our experiment. Thus, the object can be regarded as the “moving” object. Moreover, we remark that the crosstalk appeared in the recovered images is induced by the imperfect circular polarization of the generated reference beam, which however can be decreased by using the wave plates with higher quality, e.g., the true zero-order wave plates.

**Fig. 7:**
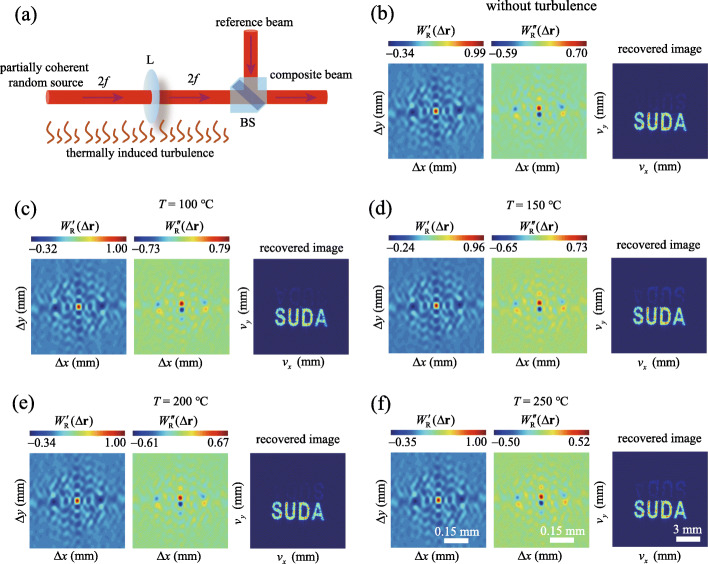
Robustness of the coherence-based encryption in the face of thermally induced turbulence modelling the atmospheric turbulence. **a** The schematics of a 2*f* imaging system in the presence of thermally induced turbulence. **b** The experimental results for the cross-spectral density of the illuminating beam and the corresponding recovered image in the turbulence free situation for reference. **c**–**f** The experimental results for the cross-spectral density of the illuminating beam and the corresponding recovered images in the presence of thermally induced turbulence with the temperature *T* of the hot graphitic plate being 100^∘^C,150^∘^C,200^∘^C, and 250^∘^C, respectively. The scale bars are shown in panel (**f**)

Finally, we demonstrate the robustness of our protocol against fluctuations in a noisy environment, such as the turbulent atmosphere. In our experiment, the fluctuations are simulated by a thermally induced turbulence generated by a hot graphitic plate with an adjustable temperature *T*. The turbulence strength grows with the temperature, which can be seen from the distortion of the intensity distribution, for example see in [40]. In the experiment, the turbulence is added only in the path of the random beam, i.e., the fluctuations are introduced into the 2*f* imaging system as shown in Fig. 7a, since the cross-spectral density measurement with the reference fields can be performed, in general, separately in a turbulence-free environment. In Fig. 7c—f, we show the experimental results for cross-spectral density and the corresponding reconstructed images for noisy environments at different temperatures. In Fig. 7b we exhibit the experimental results with no hot plate. We infer from the figures that our protocol is quite insensitive to the environmental noise. Hence, the encrypted images can be recovered despite the presence of even strong medium fluctuations. Thus, the second-order optical correlations can be regarded as a robust “lock” for our encryption system that serves to protect against attacks aimed at erasing rather than compromising the encrypted information. The robustness of the cross-spectral density distribution of light in our protocol against the environmental fluctuations is tightly linked to efficient self-healing of low-coherence light [28, 29, 40].

## Conclusions

We have presented a protocol for optical information encryption into the second-order correlations of random light fields expressed in terms of the cross-spectral density of the field. We have verified the feasibility of our protocol by carrying out a proof-of-principle experiment by encoding an optical image (plaintext) into the cross-spectral density of a random light beam with the aid of a fractional Fourier transform encoding system and decoding the plaintext through the measurement of the said cross-spectral density with the help of the recently introduced generalized Hanbury Brown–Twiss technique [40].

We argue that the vast amount of data required to reconstruct the cross-spectral density distribution, compared with a relative paucity of data necessary in the traditional encryption protocols based on measuring first-order deterministic characteristics of light fields, enhances the security of the encryption protocol. We have demonstrated experimentally that the cross-spectral density measurement is extremely robust against environmental fluctuations, e.g., the atmospheric turbulence. Therefore, the optical information can be well reconstructed despite a high level of noise in the ciphertext. This circumstance bestows certain advantages on our protocol over the alternatives in the application scenarios where an attacker aims to deliberately destroy the ciphertext rather than temper with it. Our results indicate that the spatial coherence of a random light beam can serve as a versatile tool in optical security and encryption applications. Finally, we remark that the proposed coherence-based encryption system is performed only in a very short optical path, it can, however, be extended to a long-distance optical image transmission system with the aid of the coherence phase modulation (Liu et al.: Robust far-field imaging by spatial coherence engineering, unpublished).

## Data Availability

The experimental data that support the works of this study are available from the corresponding authors on reasonable request. Declarations

## Abbreviations

DRPE: Double random phase encoding
RGGD: Rotating ground-glass disk
LP: Linear polarizer
BS: Beamsplitter
BE: Beam expander
SLM: Spatial light modulator
HWP: Half-wave plate
QWP: Quarter-wave plate
CL: Concave lens
PBS: Polarization beamsplitter
DDS: Direct-digital synthesis signal generator

## References

[1] Mandel L, Wolf E (1995). Optical Coherence and Quantum Optics.

[2] Friberg A, Setälä T (2016). Electromagnetic theory of optical coherence. J Opt Soc Am A.

[3] Chen Y, Norrman A, Ponomarenko S, Friberg A (2020). Optical coherence and electromagnetic surface waves. Prog Opt.

[4] Korotkova O, Gbur G (2020). Applications of optical coherence theory. Prog Opt.

[5] Baleine E, Dogariu A (2005). Variable coherence scattering microscopy. Phys Rev Lett.

[6] Redding B, Choma M, Cao H (2012). Speckle-free laser imaging using random laser illumination. Nat Photon.

[7] Gbur G (2014). Partially coherent beam propagation in atmospheric turbulence. J Opt Soc Am A.

[8] Ponomarenko S (2001). A class of partially coherent beams carrying optical vortices. J Opt Soc Am A.

[9] Ponomarenko S, Huang W, Cada M (2007). Dark and antidark diffraction-free beams. Opt Lett.

[10] Bogatyryova G, Fel’de C, Polyanskii P, Ponomarenko S, Soskin M, Wolf E (2003). Partially coherent vortex beams with a separable phase. Opt Lett.

[11] Gori F, Santarsiero M (2007). Devising genuine spatial correlation functions. Opt Lett.

[12] Martínez-Herrero R, Mejías P, Gori F (2009). Genuine cross-spectral densities and pseudo-modal expansions. Opt Lett.

[13] Sahin S, Korotkova O (2012). Light sources generating far fields with tunable flat profiles. Opt Lett.

[14] Mei Z, Korotkova O (2013). Random sources generating ring-shaped beams. Opt Lett.

[15] Wang F, Liu X, Yuan Y, Cai Y (2013). Experimental generation of partially coherent beams with different complex degrees of coherence. Opt Lett.

[16] Ma L, Ponomarenko S (2014). Optical coherence gratings and lattices. Opt Lett.

[17] Chen Y, Wang F, Liu L, Zhao C, Cai Y, Korotkova O (2014). Generation and propagation of a partially coherent vector beam with special correlation functions. Phys Rev A.

[18] Chen Y, Gu J, Wang F, Cai Y (2015). Self-splitting properties of a hermite-gaussian correlated schell-model beam. Phys Rev A.

[19] Sun B, Huang Z, Zhu X, Wu D, Chen Y, Wang F, Cai Y, Korotkova O (2020). Random source for generating airy-like spectral density in the far field. Opt Express.

[20] Lajunen H, Saastamoinen T (2011). Propagation characteristics of partially coherent beams with spatially varying correlations. Opt Lett.

[21] Santarsiero M, Martínez-Herrero R, Maluenda D, De Sande J, Piquero G, Gori F (2017). Partially coherent sources with circular coherence. Opt Lett.

[22] Piquero G, Santarsiero M, Martínez-Herrero R, de Sande J, Alonzo M, Gori F (2018). Partially coherent sources with radial coherence. Opt Lett.

[23] Hyde IV M, Bose-Pillai S, Wood R (2017). Synthesis of non-uniformly correlated partially coherent sources using a deformable mirror. Appl Phys Lett.

[24] Zhu X, Wang F, Zhao C, Cai Y, Ponomarenko S (2019). Experimental realization of dark and antidark diffraction-free beams. Opt Lett.

[25] Hyde M, Xiao X, Voelz D (2019). Generating electromagnetic nonuniformly correlated beams. Opt Lett.

[26] Yu J, Zhu X, Lin S, Wang F, Gbur G, Cai Y (2020). Vector partially coherent beams with prescribed non-uniform correlation structure. Opt Lett.

[27] Zhu X, Yu J, Chen Y, Wang F, Korotkova O, Cai Y (2020). Experimental synthesis of random light sources with circular coherence by digital micro-mirror device. Appl Phys Lett.

[28] Wang F, Chen Y, Liu X, Cai Y, Ponomarenko S (2016). Self-reconstruction of partially coherent light beams scattered by opaque obstacles. Opt Express.

[29] Xu Z, Liu X, Chen Y, Wang F, Liu L, Monfared Y, Ponomarenko S, Cai Y, Liang C (2020). Self-healing properties of hermite-gaussian correlated schell-model beams. Opt Express.

[30] Ding C, Koivurova M, Turunen J, Pan L (2017). Self-focusing of a partially coherent beam with circular coherence. J Opt Soc Am A.

[31] Lin S, Wang C, Zhu X, Lin R, Wang F, Gbur G, Cai Y, Yu J (2020). Propagation of radially polarized hermite non-uniformly correlated beams in a turbulent atmosphere. Opt Express.

[32] Chen Y, Ponomarenko S, Cai Y (2017). Self-steering partially coherent beams. Sci Rep.

[33] Mao H, Chen Y, Liang C, Chen L, Cai Y, Ponomarenko S (2019). Self-steering partially coherent vector beams. Opt Express.

[34] Hyde IV M, Basu S, Xiao X, Voelz D (2015). Producing any desired far-field mean irradiance pattern using a partially-coherent schell-model source. J Opt.

[35] Voelz D, Xiao X, Korotkova O (2015). Numerical modeling of schell-model beams with arbitrary far-field patterns. Opt Lett.

[36] Ma L, Ponomarenko S (2015). Free-space propagation of optical coherence lattices and periodicity reciprocity. Opt Express.

[37] Chen Y, Ponomarenko S, Cai Y (2016). Experimental generation of optical coherence lattices. Appl Phys Lett.

[38] Divitt S, Novotny L (2015). Spatial coherence of sunlight and its implications for light management in photovoltaics. Optica.

[39] Lu X, Shao Y, Zhao C, Konijnenberg S, Zhu X, Tang Y, Cai Y, Urbach H (2019). Noniterative spatially partially coherent diffractive imaging using pinhole array mask. Adv Photon.

[40] Huang Z, Chen Y, Wang F, Ponomarenko S, Cai Y (2020). Measuring complex degree of coherence of random light fields with generalized hanbury brown–twiss experiment. Phys Rev Appl.

[41] Dong Z, Huang Z, Chen Y, Wang F, Cai Y (2020). Measuring complex correlation matrix of partially coherent vector light via a generalized hanbury brown–twiss experiment. Opt Express.

[42] Xu Z, Li X, Liu X, Ponomarenko S, Cai Y, Liang C (2020). Vortex preserving statistical optical beams. Opt Express.

[43] Yang B, Chen Y, Wang F, Cai Y (2021). Trapping two types of rayleigh particles simultaneously by a focused rotational elliptical laguerre–gaussian correlated schell-model beam. J Quant Spectrosc Radiat Transf.

[44] Liu S, Guo C, Sheridan J (2014). A review of optical image encryption techniques. Opt Laser Technol.

[45] Chen W, Javidi B, Chen X (2014). Advances in optical security systems. Adv Opt Photon.

[46] Refregier P, Javidi B (1995). Optical image encryption based on input plane and fourier plane random encoding. Opt Lett.

[47] Unnikrishnan G, Joseph J, Singh K (2000). Optical encryption by double-random phase encoding in the fractional fourier domain. Opt Lett.

[48] Situ G, Zhang J (2004). Double random-phase encoding in the fresnel domain. Opt Lett.

[49] Matoba O, Javidi B (1999). Encrypted optical memory system using three-dimensional keys in the fresnel domain. Opt Lett.

[50] Rubinsztein-Dunlop H, Forbes A (2016). Roadmap on structured light. J Opt.

[51] Rosales-Guzmán C, Ndagano B, Forbes A (2018). A review of complex vector light fields and their applications. J Opt.

[52] Forbes A (2019). Structured light from lasers. Laser Photon Rev.

[53] Qu G, Yang W, Song Q, Liu Y, Qiu C-W, Han J, Tsai D-P, Xiao S (2020). Reprogrammable meta-hologram for optical encryption. Nat Commun.

[54] Trichili A, Salem A, Dudley A, Zghal M, Forbes A (2016). Encoding information using laguerre gaussian modes over free space turbulence media. Opt Lett.

[55] Fang X, Ren H, Gu M (2020). Orbital angular momentum holography for high-security encryption. Nat Photon.

[56] Qiao Z, Wan Z, Xie G, Wang J, Qian L, Fan D (2020). Multi-vortex laser enabling spatial and temporal encoding. PhotoniX.

[57] Zhao Y, Wang J (2015). High-base vector beam encoding/decoding for visible-light communications. Opt Lett.

[58] Milione G, Nguyen T, Leach J, Nolan D, Alfano R (2015). Using the nonseparability of vector beams to encode information for optical communication. Opt Lett.

[59] Xian M, Xu Y, Ouyang X, Cao Y, Lan S, Li X (2020). Segmented cylindrical vector beams for massively-encoded optical data storage. Sci Bull.

[60] Larocque H, D’Errico A, Ferrer-Garcia M, Carmi A, Cohen E, Karimi E (2020). Optical framed knots as information carriers. Nat Commun.

[61] Goodman J (2015). Statistical Optics.

[62] Ni H, Liang C, Wang F, Chen Y, Ponomarenko S, Cai Y (2020). Non-gaussian statistics of partially coherent light in atmospheric turbulence. Chin Phys B.

[63] Alfalou A, Brosseau C (2009). Optical image compression and encryption methods. Adv Opt Photon.

[64] Javidi B, Carnicer A, Yamaguchi M (2016). Roadmap on optical security. J Opt.

[65] Takeda M, Wang W, Naik D, Singh R (2014). Spatial statistical optics and spatial correlation holography: a review. Opt Rev.

